# Identifying Parkinson’s disease subtypes with motor and non-motor symptoms via model-based multi-partition clustering

**DOI:** 10.1038/s41598-021-03118-w

**Published:** 2021-12-08

**Authors:** Fernando Rodriguez-Sanchez, Carmen Rodriguez-Blazquez, Concha Bielza, Pedro Larrañaga, Daniel Weintraub, Pablo Martinez-Martin, Alexandra Rizos, Anette Schrag, K. Ray Chaudhuri

**Affiliations:** 1 Computational Intelligence Group, Universidad Politécnica de Madrid, Madrid, Spain; 2 National Center of Epidemiology, Carlos III Institute of Health, Madrid, Spain; 3 Center for Networked Biomedical Research in Neurodegenerative Diseases (CIBERNED), Carlos III Institute of Health, Madrid, Spain; 4 Departments of Psychiatry and Neurology, Perelman School of Medicine at the University of Pennsylvania, Philadelphia, USA; 5 King’s College London, Department of Neurosciences, Institute of Psychiatry, Psychology & Neuroscience and Parkinson’s Foundation Centre of Excellence, King’s College Hospital, London, UK; 6 Department of Clinical and Movement Neurosciences, UCL Queen Square Institute of Neurology, University College London, London, UK

**Keywords:** Movement disorders, Parkinson's disease

## Abstract

Identification of Parkinson’s disease subtypes may help understand underlying disease mechanisms and provide personalized management. Although clustering methods have been previously used for subtyping, they have reported generic subtypes of limited relevance in real life practice because patients do not always fit into a single category. The aim of this study was to identify new subtypes assuming that patients could be grouped differently according to certain sets of related symptoms. To this purpose, a novel model-based multi-partition clustering method was applied on data from an international, multi-center, cross-sectional study of 402 Parkinson’s disease patients. Both motor and non-motor symptoms were considered. As a result, eight sets of related symptoms were identified. Each of them provided a different way to group patients: impulse control issues, overall non-motor symptoms, presence of dyskinesias and pyschosis, fatigue, axial symptoms and motor fluctuations, autonomic dysfunction, depression, and excessive sweating. Each of these groups could be seen as a subtype of the disease. Significant differences between subtypes (*P*< 0.01) were found in sex, age, age of onset, disease duration, Hoehn & Yahr stage, and treatment. Independent confirmation of these results could have implications for the clinical management of Parkinson’s disease patients.

## Introduction

Parkinson’s disease (PD) is a progressive neurodegenerative disease that is clinically characterized by a broad spectrum of motor and non-motor manifestations^1^. There is, however, considerable clinical phenotypic and natural history related variability between PD patients, which may indicate the existence of disease subtypes. Identification of PD subtypes may help understand the underlying disease mechanisms, since homogeneous groups of patients may be more likely to share pathological and genetic features. In addition, identification of PD subtypes may ultimately lead to more precise treatment strategies (i.e., precision medicine)^2^.

Data-driven techniques such as clustering may be suitable for establishing PD subtypes. In clustering, patients are assigned to several groups (i.e., clusters) so that patients belonging to the same group share similarities. Each of these groups is usually then considered a subtype of the disease. Previous clustering studies have already identified PD subtypes with motor and non-motor symptoms^3–8^. However, to the best of our knowledge, all of them have used single-partition clustering methods such as k-means^9^, latent class analysis^10^, Gaussian mixture model^11^, agglomerative hierarchical clustering^12^. Single-partition clustering algorithms assume the existence of a single true clustering in a dataset. As a result, each patient is assigned to a single subtype that is defined by all the considered symptoms.

Recently, several issues have been raised about data-driven PD subtypes, such as the low number in the samples, their lack of internal homogeneity, and their difficulty to reproduce meaningful data in real life and external validity^13,14^. We believe that these issues may be a consequence of using single-partition clustering methods. The assumption that each patient should be assigned to a single generic subtype does not hold for PD, which is usually multifaceted and can be meaningfully partitioned in multiple ways^15,16^. For this reason, we advocate for model-based multi-partition clustering^17–20^, which extends model-based clustering^11^ by producing mixture models with multiple categorical latent variables. The idea is to use statistical principles to find sets of related symptoms where patients are divided into a number of distinct groups. Each set of symptoms defines a different clustering of patients. As a result, each patient is assigned to one subtype for each clustering. The analysis of these subtypes and their associations may provide more accurate insights about the considered symptoms, as well as their relationship with socio-demographic and clinical information of the patients.

Based on the above, the objectives of our study were: (i) to identify PD subtypes using model-based multi-partition clustering, and (ii) to analyze the associations between the resulting subtypes. To this end, we developed a novel model-based multi-partition clustering algorithm, and applied this method on data from a large, multi-center, international, and well-characterized cohort of patients.

## Methods

### Data

The analysis was carried out on data gathered from the first validation study of the Movement Disorder Society Non-Motor Rating Scale (MDS-NMS), an international, multi-center, cross-sectional study that included PD English-speaking patients from England and the United States^21^. The study was approved by the institutional review boards or ethics committees of the participating centers. All patients gave their written informed consent to participate in the study. Institutional review boards or ethics committees that approved the study: (1) National Research Ethics Service Committee East Midlands-Northampton, England; (2) Institutional Review Board at the Perelman School of Medicine at the University of Pennsylvania, United States. In addition, the study was conducted according to good clinical practice and all research was performed in accordance with relevant guidelines and regulations. Data are publicly available in our GitHub repository^22^. For all patients, socio-demographic information and basic clinical variables (i.e., sex, age, age of onset, and disease duration) were recorded and the following assessments were applied:

1. The Movement Disorder Society Unified Parkinson’s Disease Rating Scale (MDS-UPDRS)^23^, which is composed of 65 items divided across 4 parts, namely, Part I: Non-motor Experiences of Daily Living (13 items); Part II: Motor Experiences of Daily Living (13 items); Part III: Motor Examination (33 items); and Part IV: Motor Complications (6 items). Each item has 5 options of response, running from 0 (normal) to 4 (maximum intensity). The total score of each part is obtained by summing the respective item scores.
2. The MDS-NMS^21^, which is composed of 52 items grouped into 13 domains: depression, anxiety, apathy, psychosis, impulse control and related disorders (ICRDs), cognition, orthostatic hypotension, urinary, sexual, gastrointestinal, sleep and wakefulness, pain, and other. Each item is scored for both frequency and severity, where both scores have 5 options of response, ranging from 0 (normal) to 4 (maximum intensity). Each item score is generated by multiplying frequency and severity. The score of each domain is obtained by summing the respective item scores. The MDS-NMS also includes a subscale for non-motor fluctuations, composed of 8 items, which was not considered in this study.
3. The Hoehn & Yahr (H&Y) staging system^24^, which ranges from 1 to 5.

Motor items from the MDS-UPDRS were classed as 5 motor cardinal signs: tremor, rigidity, bradykinesia, dyskinesias and motor fluctuations; and 2 motor subtypes: axial symptoms and postural instability gait difficulty (PIGD)^25^. This resulted in 7 motor variables. The specific MDS-UPDRS items that constitute each motor variable are provided in Section 1 of the Supplementary Information. Additionally, items from the MDS-NMS were grouped into their respective domains, with the exception of the items from the ”other” domain (unintentional weight loss, decrease in sense of smell, physical fatigue, mental fatigue, and excessive sweating). These items were individually considered due to their individual and unique status. This resulted in 17 non-motor variables. Finally, with the objective of improving the interpretability of the results, both motor and non-motor variables were normalized to the [0, 1] range using min-max scaling.

A total of 402 patients were considered for this study. Average onset age was 59 ± 11 (s.d.) years, 62% were male and average PD duration was 8 ± 6 (s.d.) years. 13% of the patients were in H&Y stage 1; 54% in H&Y stage 2; 28% in H&Y stage 3; and 5% in H&Y stage 4. No patients in this study were in H&Y stage 5. Regarding medication, 87% of the patients took levodopa. The average levodopa daily dose (LDD) for these patients was 658.57 ± 503.55 milligrams (mg). In addition, 42% of the patients received dopamine agonist (DA) treatment. The average levodopa-equivalent daily dose of DA (LEDD-DA), calculated following Tomlinson et al.^26^, for these patients was 226.84 ± 132.14 mg. Finally, with respect to missing information, 64 values (< 1% of the total) were missing, mostly in the Sexual domain of the MDS-NMS. As our multi-partition clustering method was able to work with missing information, no patients were excluded from the analysis. For more information about the data, see Table 1.

**Table 1:** Descriptive statistics of the data. Numbers between parentheses correspond to standard deviations (s.d.).

	N^1^	Mean	(s.d.)	Median	Min^2^	Max^2^
Age	402	67.42	(9.96)	68	35	93
Age of onset	402	59.23	(10.67)	59	26	93
Disease duration	402	8.19	(5.93)	7	0	35
H&Y	402	2.25	(0.74)	2	1	4
**MDS-UPDRS**						
Tremor	391	0.13	(0.12)	0.11	0.00	0.57
Rigidity	398	0.19	(0.16)	0.15	0.00	1.00
Dyskinesias	402	0.07	(0.15)	0.00	0.00	1.00
Motor fluctuations	401	0.16	(0.19)	0.06	0.00	0.81
Bradykinesia	394	0.29	(0.17)	0.25	0.00	0.89
Axial symptoms	402	0.23	(0.15)	0.21	0.00	0.86
PIGD	393	0.22	(0.19)	0.15	0.00	0.85
**MDS-NMS**						
Depression	401	0.07	(0.13)	0.01	0.00	0.90
Anxiety	402	0.09	(0.13)	0.03	0.00	0.84
Apathy	402	0.08	(0.15)	0.00	0.00	0.75
Psychosis	402	0.03	(0.06)	0.00	0.00	0.56
ICRDs	401	0.02	(0.05)	0.00	0.00	0.39
Cognition	402	0.10	(0.12)	0.05	0.00	0.69
Orthostatic hypotension	402	0.07	(0.13)	0.00	0.00	0.75
Urinary	402	0.16	(0.19)	0.08	0.00	1.00
Sexual	375	0.14	(0.25)	0.00	0.00	1.00
Gastrointestinal	401	0.10	(0.12)	0.06	0.00	0.73
Sleep and wakefulness	401	0.12	(0.12)	0.08	0.00	0.79
Pain	402	0.13	(0.15)	0.08	0.00	0.83
Unintentional weight loss	402	0.06	(0.18)	0.00	0.00	1.00
Decrease in sense of smell	402	0.39	(0.40)	0.25	0.00	1.00
Physical fatigue	402	0.21	(0.27)	0.06	0.00	1.00
Mental fatigue	402	0.09	(0.19)	0.00	0.00	1.00
Excessive sweating	402	0.07	(0.18)	0.00	0.00	1.00

^1^Sample size without missing values.

^2^Min and max recorded values.

### Model-based multi-partition clustering method

A novel model-based multi-partition clustering method was developed to identify groups of individuals with specific patterns in the motor and non-motor domains. The proposed method learned a conditional linear Gaussian Bayesian network (BN)^27^ with multiple categorical latent variables. Each latent variable provided a unique way to partition PD patients according to a unique set of symptom variables. Each group of patients was considered a PD subtype with respect to the partition variables.

Two components can be distinguished in every BN: (i) a directed acyclic graph that encodes conditional independences among triplets of variables in the BN; and (ii) a set of parameters that describe the conditional probability distributions of each variable given its parents in the graph. Together, both of these elements define a unique joint probability distribution. BNs are useful in multi-partition clustering for several reasons. First, their graphical structure allows for an easier interpretation, showing which variables define each partition, and how partitions relate to each other. Second, their conditional independences result in more compact models that are easier to learn from data. Finally, BNs allow probabilistic inference, which is useful for making predictions, diagnoses and explanations.

Our proposal iteratively explores the space of conditional linear Gaussian BNs using five latent operators and a variational Bayesian^28^ version of the structural expectation-maximization^29^ algorithm. Latent operators are tasked with introducing latent variables, removing latent variables, and changing the cardinality (i.e., number of subtypes) of latent variables. Each application of these operators produces a candidate model whose structure is refined using the variational Bayesian structural expectation-maximization algorithm. Once all the candidate models have been evaluated, the highest scoring model is selected. This process is iteratively repeated until the model score ceases to increase. Given its greedy nature, we refer to this method as greedy latent structure learner. It is formally defined in Sections 2 and 3 of the Supplementary Information, and its implementation in Java 8 is publicly available in the project’s Github repository^22^.

### Analysis of multi-partition PD subtypes

By using a conditional linear Gaussian BN, each subtype in a partition was defined by a linear Gaussian distribution whose dimensions corresponded to the partition symptoms. The symbol μ was used to denote the mean of this subtype for a specific symptom and the symbol $\sigma$ was used to denote the s.d. In addition, to improve the readability of these subtypes, we devised a simple scale that considered the quartiles of the normalized [0, 1] range to refer to the mean symptom severity: (i) slight [0.01, 0.25]; (ii) mild [0.26, 0.50]; (iii) moderate [0.51, 0.75]; and (iv) severe [0.76, 1]. Note that this scale differs from the MDS-UPDRS and MDS-NMS ratings.

To explore the relationship between socio-demographic information, basic clinical variables, H&Y stage and the identified subtypes, hypothesis tests were performed. Each pair of subtypes in a partition were compared. For continuous variables such as age, age of PD onset, PD duration, LDD, and LEDD-DA, an ANOVA test or a Mann-Whitney U-test (both implemented in the Python library SciPy version 1.5.2) was used. When three or more groups were present in a clustering, an ANOVA test or a Kruskal-Wallis test (both implemented in SciPy) was performed instead, followed by a post-hoc analysis using Tukey’s range test (implemented in the Python library Statsmodels version 0.11.1). For categorical variables such as the sex of the patient, the presence of levodopa and DA treatments, and discrete variables such as the H&Y stage, $\chi ^{2}$ tests (implemented in SciPy) were performed. Statistical significance was defined as *p*-value *p* < 0.01.

To analyze the associations between the identified subtypes, probabilistic inference was employed. For example, consider a hypothetical multi-partition clustering model with two partitions, *A* and *B*, which are connected by an arc in the model. Partition *A* defines two PD subtypes {*A*1, *A*2} according to a set of symptoms. Partition *B* defines three PD subtypes {*B*1, *B*2, *B*3} according to a different set of symptoms. We are interested in estimating the difference between the probability distributions *P*(*B*) and *P*(*B*|*A* = *A*1), but also the difference between *P*(*B*) and *P*(*B*|*A* = *A*2). That is, how being assigned a subtype in *A* affects the probability distribution of *B*. The inverse probabilistic queries are also relevant (i.e., how being assigned a subtype in *B* affects the probability distribution of *A*). Since each subtype in a partition is characterized by a set of symptoms with a certain severity, we are incidentally studying the relationships between their respective symptoms (i.e., how an increase or decrease of the severity of certain symptoms affect the probability of suffering the other symptoms with more or less severity) when we analyze the relationships between subtypes of different partitions. In this study, probabilistic queries were carried out using Monte Carlo sampling in the tool for BN analysis GeNIe (version 3.0).

## Results

### Multi-partition PD subtypes

**Figure 1:**
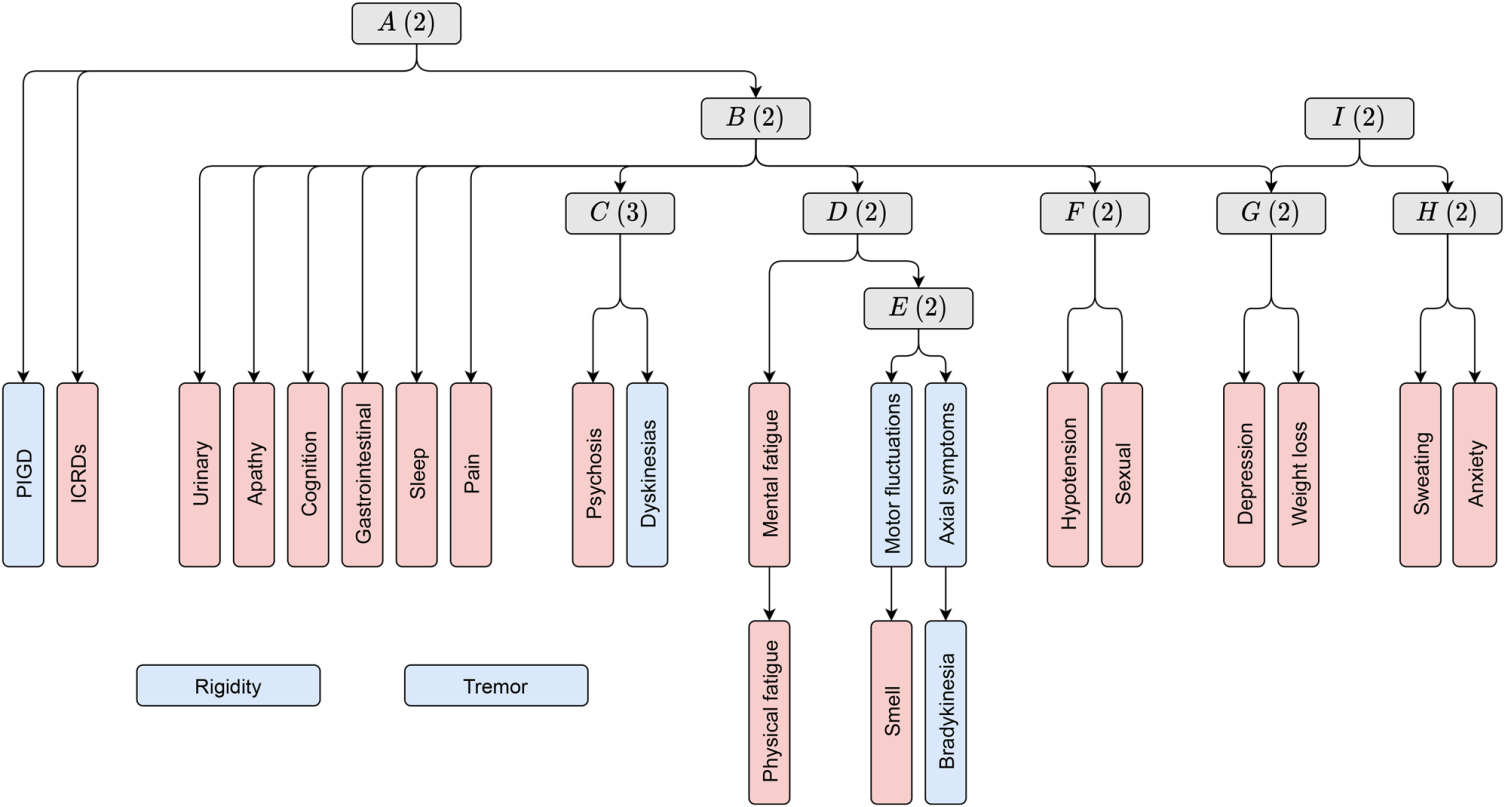
BN structure of the multi-partition clustering model. Blue nodes represent motor variables and red nodes represent non-motor variables. Grey nodes represent categorical latent variables (i.e., partitions), where the number in parentheses corresponds to the variable’s cardinality (i.e., the number of subtypes). Graph arcs represent conditional dependences.

The BN structure that resulted from applying our multi-partition clustering algorithm on the 7 motor and 17 non-motor variables is portrayed in Fig. 1. It consisted of 9 (alphabetically-named) latent variables. Each latent variable defined a unique partition according its descendant symptom variables in the graph. For example, in partition *A*, patients were divided into two subtypes according to the severity of their ICRDs and PIGD. There was, however, one latent variable that differed from the rest by not being directly related to any symptom variable. Instead, it acted as an auxiliary latent variable that connected partitions *G* (weight loss-depression) and *H* (excessive sweating-anxiety). This variable was *I*, and to simplify the analysis, its relevant information was condensed in those of *G* and *H*. As a result, 8 partitions were discovered, each with a different number of subtypes. The sex, age, age of onset, disease duration, and H&Y stage of each subtype is provided in Table 2. In addition, treatment information of each subtype is provided in Table 3. Significant differences between subtypes are included. Results (i.e., *p*-values) of the statistical tests that were performed are provided in Section 4 of the Supplementary Information.

**Table 2:** Sex, age, age of onset, disease duration and H&Y stage of each PD subtype. Numbers between parentheses correspond to standard deviations.

	Subtype	Sex (% male)	Age		Age of onset		Duration		H&Y	
Partition *A*	*A*1	62.39	68.02	(9.85)$^{a}$	60.00	(10.82)$^{a}$	8.02	(5.95)	2.27	(0.76)
	*A*2	61.19	64.45	(10.02)$^{a}$	55.37	(9.06)$^{a}$	9.07	(5.79)	2.16	(0.62)
Partition *B*	*B*1	60.80	66.07	(10.18)$^{b}$	59.18	(10.79)	6.89	(5.15)$^{b}$	2.05	(0.71)$^{b}$
	*B*2	63.55	68.75	(9.58)$^{b}$	59.28	(10.59)	9.48	(6.36)$^{b}$	2.45	(0.72)$^{b}$
Partition *C*	*C*1	60.74	67.42	(10.19)	60.72	(10.70)$^{c_{1}, c_{2}}$	6.70	(5.35)$^{c_{1}, c_{2}}$	2.10	(0.73)$^{c_{1}, c_{2}}$
	*C*2	64.29	67.10	(9.83)	56.98	(11.00)$^{c_{1}}$	10.12	(6.19)$^{c_{1}}$	2.46	(0.70)$^{c_{1}}$
	*C*3	64.58	68.19	(9.22)	56.92	(8.37)$^{c_{2}}$	11.27	(5.73)$^{c_{2}}$	2.54	(0.71)$^{c_{2}}$
Partition *D*	*D*1	61.76	68.17	(10.21)	60.48	(10.92)$^{d}$	7.68	(5.65)$^{d}$	2.17	(0.74)
	*D*2	63.08	65.87	(9.27)	56.60	(9.66)$^{d}$	9.27	(6.36)$^{d}$	2.42	(0.72)
Partition *E*	*E*1	63.32	68.61	(9.75)$^{e}$	62.25	(10.26)$^{e}$	6.36	(5.18)$^{e}$	2.13	(0.74)$^{e}$
	*E*2	61.08	66.26	(10.05)$^{e}$	56.27	(10.26)$^{e}$	10.00	(6.08)$^{e}$	2.37	(0.72)$^{e}$
Partition *F*	*F*1	54.67$^{f}$	66.64	(10.49)	59.29	(11.04)	7.35	(5.29)$^{f}$	2.11	(0.75)$^{f}$
	*F*2	70.74$^{f}$	68.31	(9.26)	59.15	(10.27)	9.16	(6.46)$^{f}$	2.41	(0.69)$^{f}$
Partition *G*	*G*1	63.09	67.37	(10.16)	59.31	(10.86)	8.05	(5.93)	2.20	(0.74)
	*G*2	58.82	67.74	(9.24)	58.91	(10.02)	8.73	(5.93)	2.46	(0.72)
Partition *H*	*H*1	63.55	68.25	(9.67)$^{h}$	60.38	(10.31)$^{h}$	7.87	(5.99)$^{h}$	2.23	(0.76)
	*H*2	58.25	65.02	(10.44)$^{h}$	55.87	(11.06)$^{h}$	9.15	(5.67)$^{h}$	2.31	(0.69)

$^a$Significant differences between *A*1 and *A*2. $^b$Significant differences between *B*1 and *B*2. $^{c_{1}}$Significant differences between *C*1 and *C*2. $^{c_{2}}$Significant differences between *C*1 and *C*3. $^d$Significant differences between *D*1 and *D*2. $^e$Significant differences between *E*1 and *E*2. $^f$Significant differences between *F*1 and *F*2. $^h$Significant differences between *H*1 and *H*2. Statistical significance was defined as *p*-value *p* < 0.01.

#### Partition *A* (ICRDs-PIGD)

- Subtype *A*1 (83%) was characterized by 335 patients that did not show problems to control their impulses ($\mu$ = 0.00, $\sigma$ = 0.00), but did show slight PIGD ($\mu$ = 0.22, $\sigma$ = 0.19).
- Subtype *A*2 (17%) was characterized by 67 patients that showed slight problems to control their impulses ($\mu$ = 0.09, $\sigma$ = 0.08), and also presented slight PIGD ($\mu$ = 0.20, $\sigma$ = 0.16).

#### Partition *B* (apathy-cognitive-pain-gastrointestinal-sleep-urinary)

- Subtype *B*1 (49%) was formed of 199 patients that showed no apathy ($\mu$ = 0.00, $\sigma$ = 0.00), slight cognitive changes ($\mu$ = 0.03, $\sigma$ = 0.04), slight pain ($\mu$ = 0.06, $\sigma$ = 0.07), slight gastrointestinal problems ($\mu$ = 0.04, $\sigma$ = 0.05), slight sleep disorders ($\mu$ = 0.06, $\sigma$ = 0.07), and slight urinary issues ($\mu$ = 0.06, $\sigma$ = 0.09).
- Subtype *B*2 (51%) was formed of 203 patients that showed slight apathy ($\mu$ = 0.16, $\sigma$ = 0.18), slight cognitive changes ($\mu$ = 0.17, $\sigma$ = 0.14), slight pain ($\mu$ = 0.19, $\sigma$ = 0.17), slight gastrointestinal problems ($\mu$ = 0.15, $\sigma$ = 0.14), slight sleep disorders ($\mu$ = 0.17, $\sigma$ = 0.13), and slight urinary issues ($\mu$ = 0.25, $\sigma$ = 0.22).

**Table 3:** Treatment information of each subtype. Numbers between parentheses correspond to standard deviations. $^a$Significant differences between *A*1 and *A*2. $^b$Significant differences between *B*1 and *B*2. $^{c_{1}}$Significant differences between *C*1 and *C*2. $^{c_{2}}$Significant differences between *C*1 and *C*3. $^{c_{3}}$Significant differences between *C*2 and *C*3. $^d$Significant differences between *D*1 and *D*2. $^e$Significant differences between *E*1 and *E*2. $^f$Significant differences between *F*1 and *F*2. $^h$Significant differences between *H*1 and *H*2. Statistical significance was defined as *p*-value *p* < 0.01.

	Subtype	Levodopa (% medicated)	LDD (mg)		DA (% medicated)	LEDD-DA (mg)	
Partition *A*	*A*1	85.67	613.87	(451.01)$^{a}$	39.70	228.48	(129.82)
	*A*2	91.04	868.87	(665.06)$^{a}$	55.22	220.95	(141.89)
Partition *B*	*B*1	81.41$^{b}$	525.25	(351.89)$^{b}$	48.74	239.56	(129.44)
	*B*2	91.63$^{b}$	774.68	(581.99)$^{b}$	64.04	209.93	(134.68)
Partition *C*	*C*1	80.99$^{c_{1}}$	519.65	(336.50)$^{c_{1}, c_{2}}$	41.32	229.70	(136.11)
	*C*2	96.43$^{c_{1}}$	737.59	(471.80)$^{c_{1}, c_{3}}$	44.64	229.60	(153.64)
	*C*3	91.67	1083.44	(830.89)$^{c_{2}, c_{3}}$	41.67	205.60	(138.70)
Partition *D*	*D*1	83.46	588.95	(419.35)$^{d}$	41.18	236.63	(131.46)
	*D*2	93.08	789.18	(612.90)$^{d}$	44.62	207.93	(132.55)
Partition *E*	*E*1	77.89$^{e}$	510.68	(375.82)$^{e}$	34.17$^{e}$	215.56	(118.79)
	*E*2	95.07$^{e}$	777.33	(559.59)$^{e}$	50.25$^{e}$	234.36	(140.41)
Partition *F*	*F*1	83.64	599.10	(485.26)$^{f}$	44.39	223.82	(121.82)
	*F*2	89.89	721.55	(516.21)$^{f}$	39.89	230.67	(144.93)
Partition *G*	*G*1	84.54	613.47	(452.18)$^{g}$	43.85	239.25	(134.54)$^{g}$
	*G*2	94.12	809.66	(626.64) $^{g}$	36.47	171.19	(105.81)$^{g}$
Partition *H*	*H*1	84.95	593.97	(416.06)$^{h}$	42.81	236.88	(124.85)
	*H*2	91.26	833.11	(658.00)$^{h}$	40.78	196.24	(149.73)

#### Partition *C* (dyskinesias-psychosis)

- Subtype *C*1 (60%) was composed of 242 patients that showed no dyskinesias ($\mu$ = 0.00, $\sigma$ = 0.01) or psychosis ($\mu$ = 0.00, $\sigma$ = 0.00).
- Subtype *C*2 (28%) was composed of 112 patients that showed slight dyskinesias ($\mu$ = 0.18, $\sigma$ = 0.18) and slight psychosis ($\mu$ = 0.02, $\sigma$ = 0.03).
- Subtype *C*3 (12%) was composed of 48 patients that showed slight dyskinesias ($\mu$ = 0.15, $\sigma$ = 0.23) and slight psychosis ($\mu$ = 0.14, $\sigma$ = 0.11).

#### Partition *D* (mental fatigue-physical fatigue)

- Subtype *D*1 (67%) consisted of 272 patients that showed no mental fatigue ($\mu$ = 0.00, $\sigma$ = 0.01) and slight physical fatigue ($\mu$ = 0.14, $\sigma$ = 0.06).
- Subtype *D*2 (33%) consisted of 130 patients that showed mild mental fatigue ($\mu$ = 0.28, $\sigma$ = 0.23) and mild physical fatigue ($\mu$ = 0.35, $\sigma$ = 0.06).

#### Partition *E* (axial symptoms-bradykinesia-loss of smell-motor fluctuations)

- Subtype *E*1 (49%) was constituted by 199 patients that showed slight axial symptoms ($\mu$ = 0.19, $\sigma$ = 0.15), slight bradykinesia ($\mu$ = 0.21, $\sigma$ = 0.02), mild loss of smell ($\mu$ = 0.29, $\sigma$ = 0.15), but no motor fluctuations ($\mu$ = 0.00, $\sigma$ = 0.02).
- Subtype *E*2 (51%) was constituted by 203 patients that showed mild axial symptoms ($\mu$ = 0.26, $\sigma$ = 0.15), mild bradykinesia ($\mu$ = 0.30, $\sigma$ = 0.02), moderate loss of smell ($\mu$ = 0.51, $\sigma$ = 0.15), and mild motor fluctuations ($\mu$ = 0.30, $\sigma$ = 0.16).

#### Partition *F* (orthostatic hypotension-sexual problems)

- Subtype *F*1 (53%) was composed of 214 patients that showed no orthostatic hypotension ($\mu$ = 0.00, $\sigma$ = 0.02) and slight sexual problems ($\mu$ = 0.01, $\sigma$ = 0.03).
- Subtype *F*2 (47%) was composed of 188 patients that showed slight orthostatic hypotension ($\mu$ = 0.15, $\sigma$ = 0.17) and mild sexual problems ($\mu$ = 0.29, $\sigma$ = 0.30).

#### Partition *G* (weight loss-depression)

- Subtype *G*1 (79%) was characterized by 317 patients that showed no weight loss ($\mu$ = 0.00, $\sigma$ = 0.01) and slight depression ($\mu$ = 0.03, $\sigma$ = 0.04).
- Subtype *G*2 (21%) was characterized by 85 patients that showed mild weight loss ($\mu$ = 0.26, $\sigma$ = 0.32) and slight depression ($\mu$ = 0.24, $\sigma$ = 0.20).

#### Partition *H* (excessive sweating-anxiety)

- Subtype *H*1 (74%) consisted of 299 patients that showed no degree of excessive sweating ($\mu$ = 0.00, $\sigma$ = 0.01) and slight anxiety ($\mu$ = 0.06, $\sigma$ = 0.07).
- Subtype *H*2 (26%) consisted of 103 patients that showed mild degree of excessive sweating ($\mu$ = 0.27, $\sigma$ = 0.28) and slight anxiety ($\mu$ = 0.19, $\sigma$ = 0.19).

A total of 29 probabilistic queries were performed to analyze the connections between the identified subtypes. They are provided in Section 4 of the Supplementary Information.

### Comparison with other model-based clustering methods.

We compared our model-based multi-partition clustering method with two model-based single-partition clustering methods (i.e., the latent class model^10^, the Gaussian mixture model^11^, and the unsupervised *k*-dependence Bayesian classifier^30^), and two model-based multi-partition clustering methods (i.e., the Gaussian expansion simplification until termination algorithm^17^, and the multi-partition mixture model^18^). We evaluated the quality of the results from both a data fitting and a clustering perspective.

In this comparative analysis, we observed that multi-partition clustering methods were able to obtain multiple partitions from data, which resulted in a higher number of subtypes than single-partition clustering methods. These subtypes were not only more specific, but also more faithful to the data (i.e., higher model selection scores). From the considered methods, our approach returned the highest scoring model. The Gaussian expansion simplification until termination algorithm also obtained a high model selection score. However, its model suffered from overfitting and was difficult to interpret (it identified 18 partitions with 55 subtypes). For more information about the model selection process, see Section 4 of the Supplementary Information.

## Discussion

### Clinical interpretation of PD subtypes and their associations

Partitions were underpinned by a reasonable spread of contributory PD symptoms, thus bridging a statistical and clinical divide. Tremor and rigidity were the exceptions, appearing to be independent of the rest of variables in the model (see Fig. 1). Weak correlation between rigidity, tremor, and non-motor symptoms is not uncommon and has also been observed in a recent study that considered a similar population^31^.

In partition *A*, patients were divided into two subtypes according to the severity of their ICRDs and PIGD. Although the mean PIGD of the subtypes did not differ by much, subtype *A*2 was characterized by a higher severity of ICRDs, a younger age and a younger age of onset. A relationship between young age, early PD onset and more severe ICRDs has been previously observed^32^. Both socio-demographic aspects are known risk factors for ICRDs along with motor complications, a pre-PD history of ICRDs, and a DA treatment^33^. Related to this, we observed a higher percentage of DA treated patients in *A*2 than in *A*1. However, no causal relationship could be extracted from this observation.

Apathy, cognition, pain, gastrointestinal, sleep, and urinary symptoms were associated in partition *B*. Two subtypes were identified, where patients characterized as subtype *B*2 presented a higher severity of these symptoms. This subtype is consistent with the Parkinson’s apathy subtype^34,35^, which has been described to be formed of older patients that showed cognitive impairment, sleep disorders, and relatively severe motor symptoms. The relationship between sleep disorders and urinary problems may indicate the presence of nocturia^36^. In addition, a recent study has also identified a relationship between constipation and cognitive dysfunction in two independent cohorts of patients^37^.

Partition *C* distinguished three subtypes that differed according to the severity of dyskinesias and psychosis. Subtypes *C*2 and *C*3 presented a higher severity of these symptoms than subtype *C*1. In addition, patients in *C*3 showed more acute psychosis than those in *C*2. Both *C*2 and *C*3 consisted of patients with a longer duration of the disease, a younger age of onset, and a higher LDD. These subtypes coincided with the observation that dyskinesias and psychosis are usually present in late stages of PD and may be associated with higher dopaminergic treatment doses^38,39^. Moreover, as PD progresses, individuals lose their long-duration response to dopaminergic treatment, usually resulting in higher doses^40^.

Fatigue is considered a common and complex non-motor symptom of PD, prevalent from the prodromal to the palliative stage. It is usually present from early stages of the disease and may often persist or even worsen over time^41^. While fatigue is usually regarded as an independent symptom, it has been moderately associated with apathy, sleep disorders, depression, and motor problems^42,43^. Our model was able to capture this duality by identifying a specific partition for fatigue symptoms, and connecting it with partitions *B* (apathy, sleep and depression) and *E* (motor problems). In addition, patients that suffered from more severe fatigue showed a longer duration of the disease and a younger age of PD onset.

Bradykinesia, axial symptoms, and motor fluctuations were associated in partition *E* with a decrease in sense of smell (i.e., hyposmia). Patients were divided into two subtypes according to *E*. While both subtypes presented motor issues, *E*2 was characterized by a higher severity of motor symptoms, hyposmia, and the presence of motor fluctuations. Anosmia/hyposmia is considered a preclinical marker of PD with relatively static severity. However, while it has not been associated to any particular PD phenotype^44,45^, a recent study has noted that normosmic PD patients usually display better motor function than hyposmic PD patients^46^.

Partition *F* identified two subtypes based on orthostatic hypotension and sexual problems. While the rest of partitions were independent of the sex of the patient, 71% of patients in *F*2 were male, showing significant differences in the sex of the patients belonging to *F*1 and *F*2. We also observed significant differences in the H&Y stage and PD duration of these patients, reflecting the later occurrence of the autonomic features of orthostatic hypotension and sexual dysfunction^47–49^.

Weight loss and depression were associated in partition *G*. Two subtypes were identified, where *G*2 was characterized by patients with mild weight loss and depression. Loss of appetite due to depression is a known weight loss factor^50^. There were no significant differences in sex, age, age of onset or H&Y stage of the patients belonging to *G*1 and *G*2. With regard to treatment, we did observe significant differences between subtypes. More specifically, there were considerably fewer number of patients with DA treatment in *G*2 than in *G*1, and those medicated patients were taking a significantly lower LEDD-DA.

Regarding clustering *H*, anxiety was associated with excessive sweating. Anxiety was present in both the *H*1 and *H*2 subtypes, but patients in *H*2 showed a higher severity of this symptom along with a mild degree of excessive sweating. This result is consistent with a recent study^51^ in which anxiety and depression were more prominent in PD patients with hyperhidrosis.

It is important to note that none of the discovered subtypes were fully independent of each other. Belonging to a specific subtype in a partition influenced the subtype probabilities in the rest of partitions. By using probabilistic inference, we were able to study the effect of these associations on their respective symptoms. Some interesting patterns that we observed included: (i) patients with ICRDs (*A*2 subtype) had a 0.75 probability of presenting the symptoms of *B*2. This result is consistent with a recent study that has challenged the traditional concept of apathy and ICRDs as opposite symptoms^52^; (ii) patients that suffered psychosis (*C*3 subtype) had an 0.88 probability of suffering the symptoms of *B*2. The presence of visual hallucinations has been linked to sleep deprivation, cognitive impairment and depression^53,54^; and (iii) patients with mild mental and physical fatigue (*D*2 subtype) had a 0.79 probability of suffering the symptoms of *B*2 and a 0.73 probability of suffering the symptoms of *E*2. As previously discussed, fatigue has been related to the presence of apathetic symptoms, sleep disturbances, and higher H&Y stages^42,43^.

The majority of partitions were directly or indirectly influenced by *B*, which acted as a pivotal latent variable in the multi-partition clustering model. This aligns with the current observation that sleep disorders, depression, constipation, and other non-motor symptoms appear across the spectrum of patients with PD^55^.

### Limitations

This study has some limitations. Concerning the population of the study, patients were not specifically selected for this analysis, but rather for the validation of the MDS-NMS. Nonetheless, the large sample size and the high quality of the collected data will allow these results to be contrasted and compared with the results of future studies. The sample was comparatively younger than the average population of patients with PD. It is therefore possible that the results differ in those with an older age where higher rates of comorbidities exist. In addition, we did not report a control group, although our intention was not to describe the symptoms as discriminant from normal subjects. Concerning MDS-UPDRS and MDS-NMS, these scales do not consider patient treatment. It is therefore difficult to identify if symptom severity is a natural consequence of PD or if it is a consequence of medication. Moreover, the majority of patients in this study were medicated. Finally, we did not consider PD biomarkers, which could provide more information about the identified subtypes.

## Conclusion

Dividing PD patients into groups with common symptoms may help understand their underlying pathological processes. In this study, we used model-based multi-partition clustering to categorize patients according to 8 different sets of motor and/or non-motor symptoms. By using probabilistic inference, we were able to explore the associations between these subtypes and extract useful patterns. Independent confirmation of these results could allow for more precise PD treatments. In the future, it would be interesting to research how the evolution of PD throughout the years would affect these subtypes, and to which extent they could be markers of PD progression.

## Supplementary Information

Supplementary Information.

## Data Availability

All data, code and results are publicly available in our GitHub repository (https://github.com/ferjorosa/parkinson-subtypes).
