# Supplementary material for: Identifying Parkinson’s disease subtypes with motor and non-motor symptoms via model-based multi-partition clustering
Source: Sci Rep. 2021 Dec 8;11:23645. doi: 10.1038/s41598-021-03118-w (PMC8654994; doi:10.1038/s41598-021-03118-w)
Supplement: Supplementary file 1 — Supplementary Information. [file 41598_2021_3118_MOESM1_ESM.pdf]

Identifying Parkinson's disease subtypes with motor and non-motor symptoms via model-based multi-partition clustering: Supplementary information

Contents

1 Motor variables specification **2**

2 Methodological preliminaries **3**
- 2.1 Notation . . . 3
- 2.2 Multi-partition clustering with Bayesian networks . . . 3
- 2.3 Probabilistic inference with Bayesian networks . . . 5

3 Greedy latent structure learner **6**
- 3.1 Latent operators . . . 6
- 3.2 VB-SEM . . . 6
  - 3.2.1 Local VB-SEM . . . 8
- 3.3 Search procedure . . . 9
  - 3.3.1 Complexity analysis . . . 11

4 Results **13**
- 4.1 Model selection . . . 13
- 4.2 Statistical tests . . . 17
- 4.3 Probabilistic inference . . . 17

1 Motor variables specification

In this section, we provide the specification of the motor variables that were considered in the study.

Cardinal signs

- **Tremor** (MDS-UPDRS items 2.10, 3.15 - 3.18)
- **Rigidity** (MDS-UPDRS items 3.3a-e)
- **Dyskinesias** (MDS-UPDRS items 4.1, 4.2)
- **Motor fluctuations** (MDS-UPDRS items 4.3 - 4.6)
- **Bradykinesia** (MDS-UPDRS items 3.4 - 3.8, 3.14)

Motor subtypes

- **Axial symptoms** (MDS-UPDRS items 2.1 - 2.3, 3.1 - 3.2, 3.9, 3.13)
- **PIGD** (MDS-UPDRS items 2.12, 2.13, 3.10, 3.11, 3.12)

2 Methodological preliminaries

In this section, we provide methodological background for multi-partition clustering with Bayesian networks, as well as a brief introduction of probabilistic inference with Bayesian networks.

2.1 Notation

We use capital letters such as X, Y, Z, to denote variable names, and lower-case letters, such as x, y, z, to denote specific values taken by these variables. Sets of variables are indicated by bold capital letters, such as $\mathbf{X}$, $\mathbf{Y}$, $\mathbf{Z}$, and assignments of values to these variables are indicated by bold lower-case letters, such as $\mathbf{x}$, $\mathbf{y}$, $\mathbf{z}$.

2.2 Multi-partition clustering with Bayesian networks

Let $\mathcal{D}$ be a dataset with a set of N independent, identically distributed (i.i.d.) data instances with an associated set of categorical and continuous observed variables $\mathbf{X} = \{X_1, \ldots, X_m\}$. Multi-partition clustering algorithms [1]-[4] assume that data has been generated by a probability distribution $P(\mathbf{X})$ that can be expressed as a finite mixture model [5] with s categorical latent variables $\mathbf{H} = \{H_1, \ldots, H_s\}$. Each latent variable in the model represents a unique partition (i.e., clustering solution) with k_i probabilistic clusters:

$$P(\mathbf{X}) = \sum_{H_1} \cdots \sum_{H_s} P(\mathbf{H}) P(\mathbf{X}|\mathbf{H}) \, . \tag{1}$$

However, working with this model becomes cumbersome because the number of model parameters increases exponentially with respect to the number of categorical variables. Furthermore, its interpretation also becomes difficult because each partition is defined by all the observed variables, regardless of their relevance to it. It is therefore necessary to exploit the conditional independences that are present in the data. Conditional independence is a central concept to Bayesian networks (BNs) [6, 7]. When conditional independences are present, BNs produce a factorization of the joint probability distribution that substantially reduces the number of model parameters and allows to graphically represent relevant relationships between variables.

Given a set of variables $\mathbf{Y} = \{\mathbf{X}, \mathbf{H}\}$, a BN is defined by: (i) a *directed acyclic graph* $\mathcal{G}$ that comprises the structure of the network and represents the conditional independences among the variables, and (ii) a set of *parameters* $\boldsymbol{\theta}$ that represents the conditional probability distribution (CPD) of each variable $Y_i \in \mathbf{Y}$ given its parents $\mathbf{Pa}_i^{\mathcal{G}}$ in the graph. $\mathcal{B} = \{\mathcal{G}, \boldsymbol{\theta}\}$ is a BN with respect to $\mathcal{G}$ if and only if it satisfies the local Markov property, i.e., each variable is conditionally independent of its non-descendants given its parents in the graph. Hence, the joint probability distribution factorizes as

$$P(\mathbf{Y}) = \prod_i P(Y_i|\mathbf{Pa}_i^{\mathcal{G}}) \, . \tag{2}$$

Depending on the nature of the variables that are present in a BN, we can distinguish between categorical, continuous and mixed BNs. In this study, we are interested in clustering continuous data with multiple categorical latent variables. Therefore, we are going to work with mixed BNs, which are composed of categorical and continuous variables. More specifically, we are going to consider conditional linear Gaussian (CLG) BNs [8]. In a CLG BN, every categorical variable may only have categorical parents and its CPD is categorical. In addition, every continuous variable may have both categorical and continuous parents, and its CPD is a CLG. Let Y be a continuous variable where $\mathbf{C}$ are its continuous parents and $\mathbf{D}$ are its categorical parents. We say that Y follows a CLG distribution if for every assignment $\mathbf{d} \in \Omega_{\mathbf{D}}$ (where $\Omega_{\mathbf{D}}$ represents all possible joint assignments to $\mathbf{D}$) there are Gaussian parameters $\beta_{\mathbf{d},i}$ and $\sigma^2_{\mathbf{d},}$ such that

$$P(Y|\mathbf{C}, \mathbf{d}) = \mathcal{N}(\beta_{\mathbf{d},0} + \sum_i \beta_{\mathbf{d},i} C_i; \sigma^2_{\mathbf{d}}) \ . \tag{3}$$

Learning a BN from data is typically performed by a search method that approaches the learning process from a model selection perspective. Search methods define a hypothesis space of potential models, a set of operators to navigate this space, and a scoring function that measures how well the model fits the observed data [9]. When all the variables in the network are observed, the decomposability property of BN scores such as the Akaike information criterion (AIC) [10], and the Bayesian information criterion (BIC) [11] allows for efficient learning. However, in the presence of latent variables it is not feasible to efficiently learn a BN because scoring functions do not decompose.

Friedman's structural expectation-maximization (SEM) [12] is the most widely used algorithm for learning a BN in the presence of latent variables. It generalizes the expectation-maximization (EM) algorithm [13] to the problem of BN learning. In the expectation step, it uses the current model to estimate the values of latent variables and generates a completed dataset $\mathcal{D}^*$. Then, in the maximization step, it estimates the parameters and structure of the new model using the completed data. Any search method can be used in the maximization step. However, the scoring function to be maximized must be a penalized version of the log-likelihood (LL), such as AIC or BIC.

It is important to note the benefits of SEM compared to a brute-force approach: rather than re-estimating the model parameters after each structure change, the output of a single expectation step is used to perform many structure changes. At each iteration t, SEM selects the model $\mathcal{B}_{t+1}$ with the highest expected score. The expected score is computed over the completed data and it is referred to as $\text{score}(\mathcal{B}_{t+1} : \mathcal{D}^*_t)$. Its use is motivated by the following inequality:

$$\text{score}(\mathcal{B}_{t+1} : \mathcal{D}^*_t) - \text{score}(\mathcal{B}_t : \mathcal{D}^*_t) \leq \text{score}(\mathcal{B}_{t+1} : \mathcal{D}) - \text{score}(\mathcal{B}_t : \mathcal{D}) \ , \tag{4}$$

which states that a score improvement with respect to the completed data guarantees an improvement with respect to the observed data. Hence, Equation (4) ensures that the SEM algorithm converges to a local optimum without the need of using probabilistic inference in each structure change.

When a latent variable is known to exist, we can introduce it into the BN model and apply SEM. However, when performing multi-partition clustering, we do not know either

the appropriate number of latent variables, nor their respective cardinalities. Current literature [1, 2] has approached this problem by constructing the BN model one local move at a time (i.e., introducing a latent variable, removing an arc, increasing the cardinality of a latent variable, etc.). Surprisingly, not much attention has been paid to incorporate the SEM algorithm into the learning process. As a result, existing approaches limit their search of models to tree-like structures in order to reduce their computational complexities.

2.3 Probabilistic inference with Bayesian networks

BNs offer an additional advantage for multi-partition clustering. When the model is learned, it can be used for making predictions, diagnoses and explanations. To do this, the CPD of a variable (or a set of variables) of interest is computed given the values of other variables. This process is known as probabilistic inference.

Given a BN $\mathcal{B}$ over a set of random variables $\mathbf{X}$, probabilistic inference allows us to answer general queries of the form $P(\mathbf{I} = \mathbf{i}|\mathbf{E} = \mathbf{e})$, where $\mathbf{e}$ are the values of the evidence variables $\mathbf{E} \subset \mathbf{X}$ and $\mathbf{i}$ are the values of the variables of interest $\mathbf{I} \subseteq \{\mathbf{X} \setminus \mathbf{E}\}$, which we do not know. For example, in the medical domain, we can query the probability of a certain disease given the observed symptoms. The goal of inference can be formulated as follows:

$$P(\mathbf{I} = \mathbf{i}|\mathbf{E} = \mathbf{e}) = \frac{P(\mathbf{I} = \mathbf{i}, \mathbf{E} = \mathbf{e})}{P(\mathbf{E} = \mathbf{e})} \, .$$

The general problem of probabilistic inference in BNs was first tackled by Kim and Pearl [14]. On a very high level, inference algorithms can be divided into two main classes: exact inference methods and approximate inference methods. In this paper, we use approximate inference methods because exact inference in conditional linear Gaussian BNs is usually unfeasible [15]. For additional information on this topic, Salmeron et al. [16] provide a recent review of the literature.

3 Greedy latent structure learner

In this section, we develop a multi-partition clustering method that incorporates a variational Bayesian (VB) [17] version of SEM to learn CLG BNs with categorical latent variables from continuous data. Our method iteratively explores this space of models using five latent operators and the VB-SEM algorithm. Latent operators are tasked with introducing latent variables, removing latent variables, and changing the cardinality of latent variables. Each application of the latent operators produces a candidate model whose structure is subsequently refined using a local run of VB-SEM, which introduces, removes, or reverses BN arcs. Both aspects of the structure search (latent variables and BN arcs) are separated to reduce the computational cost of the algorithm.

We start with a description of the latent operators in Section 3.1. Then, in Section 3.2, we present the VB-SEM algorithm and its local variant (see Section 3.2.1). Finally, in Section 3.3, we discuss the search procedure.

3.1 Latent operators

- **Latent variable introduction** (LI) generates a new model by introducing a new categorical latent variable H as the parent of two variables (observed or latent) that currently have no parents. The cardinality of H is set to two. We only consider pairs of variables to reduce the computational complexity of this operator. This restriction will be compensated by the local VB-SEM algorithm (see Section 3.2.1).

- **Conditional latent variable introduction** (CLI) produces a new model by introducing a new latent variable H' as the parent of two variables (observed or latent) that currently have a latent variable H as their parent. H' becomes the new child of H. The cardinality of H' is set equal to the cardinality of H. This operator is not applicable if H only has two children. Supplementary Figure S1 provides an example application of this operator.

- **Latent variable elimination** (LE) generates a new model by removing a latent variable and its associated arcs.

- **Cardinality increase** (CI) creates a new model by increasing the cardinality of a categorical latent variable H by one. This operator is not applicable if the variable has a cardinality equal to k_{max}, which is established by the user.

- **Cardinality decrease** (CD) produces a new model by decreasing the cardinality of a categorical latent variable H by one. This operator is not applicable if H already has a cardinality of two.

3.2 VB-SEM

In this section, we introduce a variant of SEM that it is framed within the VB framework to ensure its applicability to CLG BNs. We refer to this method as VB-SEM. The introduction of the VB framework arises from the need to apply probabilistic inference in

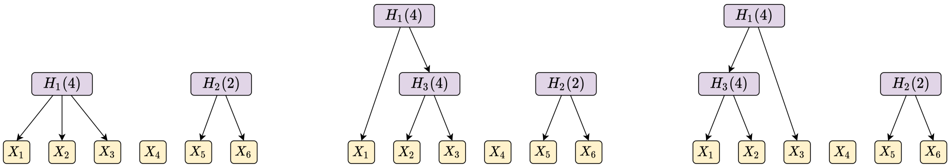

(base) (CLI candidate 1) (CLI candidate 2)

SUPPLEMENTARY FIGURE S1: Example application of the CLI operator. The base model contains two latent variables (i.e., H_1 and H_2). However only H_1 has more than two children. As a result, two candidate models are generated by introducing a new latent variable H_3 as the child of H_1. Categorical variables are colored purple while continuous variables are colored yellow. The number in parentheses accompanying each latent variable represents its cardinality.

Algorithm 1: VB-SEM

Input : $\mathcal{D}$, $\mathcal{B}_0$, $\mathcal{R}_A$

1 **for** $t = 0, 1, \ldots$ **do**

  `/* Expectation step */`

2   $\mathcal{D}_t^* \leftarrow$ Complete latent data using variational inference with $\mathcal{B}_t$

  `/* Maximization step */`

3   $\mathcal{G}_{t+1} \leftarrow$ GS($\mathcal{D}_t^*$, $\mathcal{R}_A$)

4   $\boldsymbol{\theta}_{t+1} \leftarrow$ VB-EM($\mathcal{D}$, $\mathcal{G}_{t+1}$)

5   $\mathcal{B}_{t+1} \leftarrow \{\mathcal{G}_{t+1}, \boldsymbol{\theta}_{t+1}\}$

6   **if** *score*$(\mathcal{B}_{t+1} : \mathcal{D}) \leq$ *score*$(\mathcal{B}_t : \mathcal{D})$ **then**

7   **break** `/* Stop the loop */`

Output: The resulting model $\mathcal{B}_t$

---

the expectation step of SEM. Since exact inference is NP-hard for CLG BNs (even when restricting the BN structure to a polytree) [15], approximate inference is required. Two of the most popular approximation schemes are Markov chain Monte Carlo (MCMC) [18] and variational inference (VI) [19]. We chose VI (and more specifically the VB framework) due to its speed advantage over MCMC and its easy integration with BNs through the variational message passing algorithm [20].

Algorithm 1 describes VB-SEM, which performs as follows. It receives a dataset $\mathcal{D}$, an initial model $\mathcal{B}_0$, and a set of arc restrictions $\mathcal{R}_A$ that may limit the structures produced by the algorithm (e.g., we may not be interested in adding/removing certain graph arcs). Line 1 is the main loop of the algorithm. Line 2 describes the expectation step, in which the current model $\mathcal{B}_t$ estimates the values of its latent variables and generates a completed dataset $\mathcal{D}_t^*$. Lines 3-7 describe the maximization step. In line 3, the completed dataset is used to learn the structure $\mathcal{G}_{t+1}$ of the new model $\mathcal{B}_{t+1}$. For simplicity, we use a greedy search (GS) method with arc addition, arc removal, and arc reversal operators [21]. Once the structure has been learned, the parameters $\boldsymbol{\theta}_{t+1}$ of the new model are estimated using

the observed data $\mathcal{D}$ (line 4). To this purpose, the VB-EM algorithm [22] is employed. Finally, the resultant model $\mathcal{B}_{t+1}$ is compared with the current best model $\mathcal{B}_t$, and the model with greater score with respect to $\mathcal{D}$ is selected.

VB-SEM estimates the parameters and score of the new model with respect to the observed data (i.e., the observed score) rather than with the completed data (i.e., the expected score) to improve its model fitting with respect to the observed data. Despite the desirable properties of SEM, Equation (4) offers no guarantee that the model selected at the end of the search is near the optimum of the observed score [7]. For example, if a search method is used inside SEM, only the first structure change (e.g., an arc addition) guarantees an improvement with respect to the observed score. Benjumeda et al. [23] address this problem by estimating the observed score after each structure change at the expense of the score decomposition. Their approach applies to categorical data, where exact inference can be made tractable by bounding the treewidth of the BN [24]. We instead consider the approximation of estimating the expected score when learning the BN structure, and the observed score when learning the parameters.

The scoring function that is traditionally used within the VB framework is the evidence lower bound (ELBO). The ELBO function evaluates how closely the variational distribution $Q(\mathbf{H}, \boldsymbol{\theta})$ approximates the posterior distribution of latent variables and parameters $P(\mathbf{H}, \boldsymbol{\theta}|\mathcal{D}, \mathcal{G})$ using the Kullback-Leibler (KL) divergence:

$$\text{ELBO}(\mathcal{B} : \mathcal{D}) = \log P(\mathcal{D}|\mathcal{G}) - \text{KL}(Q(\mathbf{H}, \boldsymbol{\theta})||P(\mathbf{H}, \boldsymbol{\theta}|\mathcal{D}, \mathcal{G})) \,, \tag{5}$$

where $\log P(\mathcal{D}|\mathcal{G})$ is the logarithm of the evidence (i.e., the marginal likelihood). However, when applied to models with categorical latent variables, an adjustment must be made to the ELBO in order to account for the model parameters' lack of identifiability. A common solution involves introducing a small penalty in the ELBO that considers the cardinality k_i of each latent variable H_i in the model [25]. This results in a penalized version of the ELBO (p-ELBO), which we use as our scoring function:

$$\text{p-ELBO}(\mathcal{B} : \mathcal{D}) = \text{ELBO}(\mathcal{B} : \mathcal{D}) - \sum_{i=1}^{s} \log k_i! \,. \tag{6}$$

Finally, a key aspect for the performance of VB-SEM is prior specification. When expert information is available, prior parameters can be selected to best reflect the expert knowledge. When this information is unavailable, we propose to use the following strategy: (i) observed variables assume empirical Bayes [26] priors (with maximum likelihood estimates as the values of prior parameters), and (ii) latent variables assume a symmetric Dirichlet prior with a total concentration of 1.00.

3.2.1 Local VB-SEM

Repeatedly evaluating candidate models produced by the latent operators can become prohibitive when each evaluation involves a full execution of VB-SEM. Therefore, we propose replacing VB-SEM with a local version of this method, which only considers the section of the model that is affected by the latent operator while the rest of the model is kept unchanged. We refer to this method as local VB-SEM.

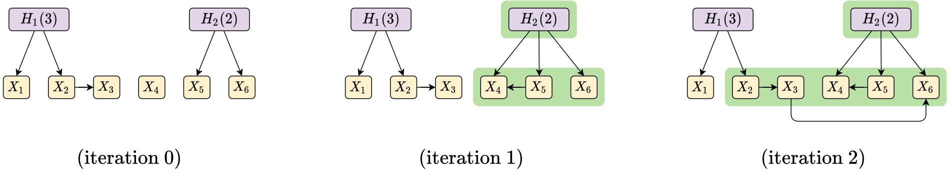

SUPPLEMENTARY FIGURE S2: Example execution of the local VB-SEM algorithm with two iterations. The initial model is a candidate of the latent introduction operator, which has introduced H_2. It introduces two arcs in its first iteration ($H_2 \rightarrow X_4$ and $X_5 \rightarrow X_4$) and one arc in its second iteration ($X_3 \rightarrow X_6$). Variables highlighted in green have their parameters estimated by the local VB-EM algorithm. Variable colors and parentheses have the same meaning as in Supplementary Figure S1.

In the local VB-SEM algorithm, we allow adding, removing, or reversing arcs that contain any of the variables considered by the latent operator. Once the structure has been learned, the local VB-EM algorithm [27] estimates the parameters of those variables belonging to the Markov blankets (MBs) of: (i) variables initially selected by the latent operator, and (ii) variables affected by a structure change (e.g., a new incoming arc). For any variable in a BN, its MB consists of the set of all its parents, children, and spouses (parents of children) in the network. Therefore, the pseudocode of local VB-SEM is identical to the pseudocode of VB-SEM (Algorithm 1), except that the structure learning process is always restrained by a specific set of arc restrictions, and the parameter learning process is done with the local VB-EM algorithm.

One iteration of local VB-SEM is computationally much cheaper than one iteration of VB-SEM because it considers fewer structure changes and it updates fewer model parameters. This also implies that a run of local VB-SEM usually requires fewer steps to converge than a run of VB-SEM. The computational complexity of local VB-SEM is upper-bounded by the computational complexity of VB-SEM.

Supplementary Figure S2 provides an example execution of the local VB-SEM algorithm. The initial model is a candidate of the LI operator, which has introduced a new latent variable H_2 as the parent of the observed variables X_5 and X_6. In its first iteration, local VB-SEM introduces two new arcs: $H_2 \rightarrow X_4$ and $X_5 \rightarrow X_4$. After the structure learning process, the local VB-EM algorithm estimates the parameters of H_2, X_4, X_5, and X_6 (highlighted in green in the figure), all of which belong to the MBs of the variables initially selected by the latent operator. In its second and last iteration, local VB-SEM introduces a new arc from X_3 to X_6 and estimates the parameters of H_2, X_2, X_3, X_4, X_5, and X_6. While X_2 does not belong to the MB of the variables initially considered by the latent operator, it does belong to the MB of a variable affected by a structure change (i.e., X_3). This is not the case of H_1 and X_1, whose parameters are kept unchanged.

3.3 Search procedure

The search starts with an initial model $\mathcal{B}_0$ established by the user. Given this model, let $\mathbf{W}$ denote the set of variables that are considered by the LI operator (i.e., the set of

Algorithm 2: Greedy latent structure learner (GLSL)

Input : $\mathcal{D}$, $\mathcal{B}_0$, k_{max}, $\mathcal{R}_A$

$\mathcal{B} \leftarrow \mathcal{B}_0$
Let $\mathbf{W}$ be the set of variables that currently have no parent in $\mathcal{B}$
Let $\mathbf{H}_C$ be the set of latent variables with 3 or more children in $\mathcal{B}$
while *True* **do**
  $\mathbf{B}_{LI} \leftarrow$ LI($\mathcal{B}$, $\mathbf{W}$, $\mathcal{D}$, $\mathcal{R}_A$)
  $\mathbf{B}_{CLI} \leftarrow$ CLI($\mathcal{B}$, $\mathbf{H}_C$, $\mathcal{D}$, $\mathcal{R}_A$)
  $\mathbf{B}_{LE} \leftarrow$ LE($\mathcal{B}$, $\mathcal{D}$, $\mathcal{R}_A$)
  $\mathbf{B}_{CI} \leftarrow$ CI($\mathcal{B}$, $\mathcal{D}$, k_{max}, $\mathcal{R}_A$)
  $\mathbf{B}_{CD} \leftarrow$ CD($\mathcal{B}$, $\mathcal{D}$, $\mathcal{R}_A$)
  $\mathcal{B}' \leftarrow$ highest scoring model in $\{\mathbf{B}_{LI}, \mathbf{B}_{CLI}, \mathbf{B}_{LE}, \mathbf{B}_{CI}, \mathbf{B}_{CD}\}$
  if $score(\mathcal{B}' : \mathcal{D}) > score(\mathcal{B} : \mathcal{D})$ **then**
    $\mathcal{B} \leftarrow \mathcal{B}'$
    Update $\mathbf{W}$ and $\mathbf{H}_C$ with respect to $\mathcal{B}$
  else
    break `/* Stop the loop */`

`/* Model refinement */`
$\mathcal{B}' \rightarrow$ VB-SEM($\mathcal{D}$, $\mathcal{B}$, $\mathcal{R}_A$)

Output: The resulting model $\mathcal{B}'$

variables that currently have no parents) and let $\mathbf{H}_C$ denote the set of latent variables that are considered by the CLI operator (i.e., the set of latent variables that currently have three or more children). Once the initial model has been established, the search method traverses the space of models by iteratively applying the latent operators. Each application of a latent operator produces a set of candidate models that are evaluated using the local VB-SEM algorithm with p-ELBO as the scoring function (see Equation (6)). Once all candidate models have been evaluated, the highest scoring model $\mathcal{B}'$ is selected. If its score is higher than that of $\mathcal{B}$, the new model takes its place and the process is repeated. Once the search stops, the resulting model is refined using a full run of VB-SEM. This search procedure is formally defined in Algorithm 2.

Supplementary Figure S3 provides an example execution of the GLSL algorithm. It starts with an empty graph with three categorical variables $\{X_1, X_2, X_5\}$ and three continuous variables $\{X_3, X_4, X_6\}$. In its first iteration, the LI operator is selected and a new latent variable H_1 is introduced as the parent of X_3 and X_4. In addition, the local run of VB-SEM results in other three arcs: $H_1 \rightarrow X_1$, $H_1 \rightarrow X_2$, and $X_5 \rightarrow X_4$. In its second iteration, the CI operator is selected, and the cardinality of H_1 is increased. No structure changes are introduced by the local VB-SEM. In its third iteration, the CLI operator is selected. A new latent variable H_2 is introduced as the child of H_1, and as the parent of X_3 and X_4. In the fourth and last iteration, the CD operator is selected, and the cardinality of H_2 is decreased. In addition, the local run of VB-SEM introduces a new arc from X_1 to X_3. Finally, the refinement run of VB-SEM introduces a new arc from X_5 to X_6 and the model is returned.

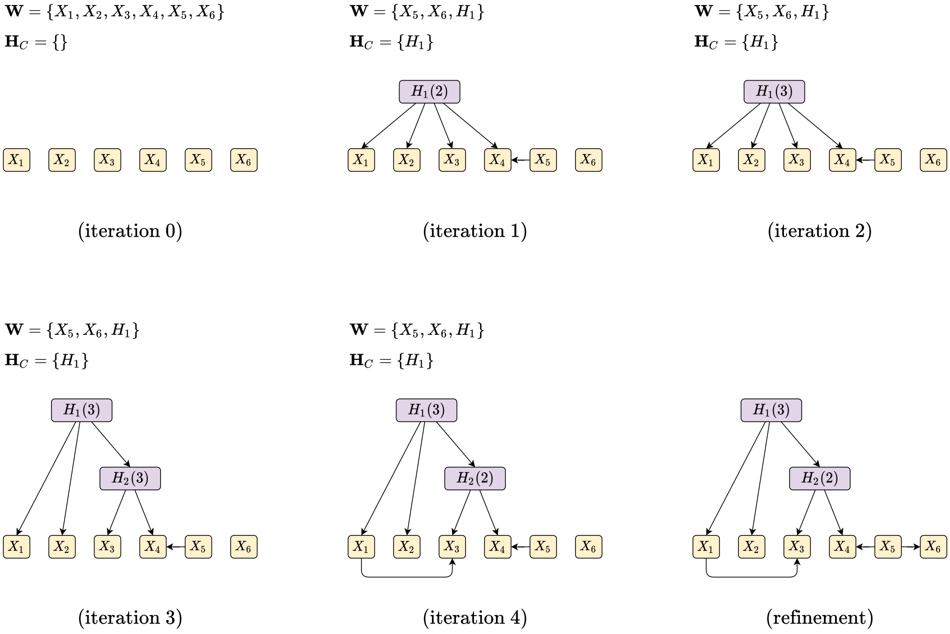

SUPPLEMENTARY FIGURE S3: Example execution of the GLSL algorithm with four iterations. It introduces a latent variable H_1 in the first iteration, it increases the cardinality of H_1 in the second iteration, it introduces a new conditional latent variable H_2 in the third iteration, and it decreases the cardinality of H_2 in the fourth iteration. Each iteration is followed by a local run of the VB-SEM algorithm, which is tasked with introducing, removing, or reversing BN arcs. Once the iteration process of GLSL finishes, a full run of VB-SEM refines the structure, introducing a new arc from X_5 to X_6. Variable colors and parentheses have the same meaning as in Supplementary Figure S1.

3.3.1 Complexity analysis

The following auxiliary variables are considered for the complexity analysis of GLSL:

- $\mathbf{W} \rightarrow$ current set of variables with no parents.
- $\mathbf{H} \rightarrow$ current set of latent variables.
- $\mathbf{H}_C \rightarrow$ current set of latent variables with three or more children.
- $\mathbf{Ch}_{Ci} \rightarrow$ current set of children variables of the latent variable $H_i \in \mathbf{H}_C$.

At each iteration of the GLSL algorithm, the CI, CD, and LE operators evaluate a total of $O(|\mathbf{H}|)$ candidate models each. In turn, the LI operator evaluates a total of $O((|\mathbf{W}|^2 - |\mathbf{W}|)/2)$ candidate models. Estimating the number of candidate models that are evaluated by the CLI operator is more complex than for LI. This is because CLI depends on the current number of latent variables and their respective number of children variables. The CLI operator evaluates a total of $O(\sum_i (|\mathbf{Ch}_{Ci}|^2 - |\mathbf{Ch}_{Ci}|)/2)$ candidate models.

Therefore, the total number of candidate models evaluated at each iteration of GLSL is $O(3|\mathbf{H}| + (|\mathbf{W}|^2 - |\mathbf{W}|)/2 + \sum_i (|\mathbf{Ch}_{Ci}|^2 - |\mathbf{Ch}_{Ci}|)/2)$. Note that each evaluation requires running the local VB-SEM algorithm.

4 Results

4.1 Model selection

In this section, we present the results of comparing GLSL multi-partition clustering model with others from the state of the art. Due to the absence of prior information, we considered empirical Bayes priors for GLSL. We compared GLSL with several model-based clustering methods from the literature that allow continuous data. We considered three single-partition clustering methods (i.e., traditional model-based clustering methods that learn a mixture model with a single latent variable) and two multi-partition clustering methods:

- **Latent class model** (LCM) [28], which learns a mixture model where all the observed variables are conditionally independent given a categorical latent variable. The cardinality of the latent variable is iteratively estimated by maximizing the BIC score. The implementation is provided in our public repository.

- **Unsupervised k-dependence Bayesian classifier** (uk-DB) [29], which learns a BN model with a single categorical latent variable that is the parent of all the observed variables. Observed variables may also be the children of other $k-1$ observed variables. The cardinality of the latent variable is iteratively estimated using a scoring function and the arcs between observed variables are usually estimated using SEM. Given the presence of mixed data, we used the p-ELBO and the VB-SEM algorithm for the structure learning process. We also considered $k = 3$. The implementation is provided in our public repository.

- **Gaussian mixture model** (GMM) [5], which learns a mixture model where all the observed variables follow a joint Gaussian distribution, and whose parameters depend on the value of the categorical latent variable. The cardinality of the latent variable is iteratively estimated by maximizing the BIC score. The implementation is provided in our public repository.

- **Gaussian expansion adjustment simplification until termination** (GEAST) [1], which learns a pouch latent tree model, where each internal node represents a latent variable, and each leaf node represents a set of observed variables. Each set of observed variables is conditionally independent of the rest of variables in the model given a categorical latent variable. Similar to a GMM, each set of observed variables follow a joint Gaussian distribution whose parameters depend on the value of the categorical latent variable. We used the original Java implementation (https://github.com/kmpoon/pltm-east). For reproducibility purposes, the implementation is also provided in our public repository.

- **Multi-partition mixture model** (MPMM) [2], which learns a mixture model with several categorical latent variables. Each latent variable is the parent of a unique subset of observed variables. Each observed variable is conditionally independent of the rest of variables given its latent parent. The implementation is provided in our public repository.

SUPPLEMENTARY TABLE S1
Models considered in this study.

Method	Log-Likelihood[1]	BIC[1]	Learning time (s)	#Partitions	#Subtypes
LCM	8808.38	8076.81	40.34	1	5
GMM	8795.14	6849.29	79.26	1	2
uk-DB	7717.87	7277.13	115.75	1	2
GEAST	11150.21	10397.66	23650.34	18	55
MPMM	5104.10	4912.21	1216.89	1	2
GLSL-CIL	11028.08	10635.31	7764.83	9	19

[1] The higher the score the better.

Abbreviations: LL, log-likelihood; BIC, Bayesian information criterion; s, seconds; #Partitions, number of partitions; #Subtypes, total number of subtypes across all partitions; #Parameters, number of model parameters.

From the considered methods, only GLSL was able to inherently work with missing values. For this reason, the rest of methods worked with an imputed version of the dataset. Missing data imputation was carried out by the multiple imputation by chained equations (MICE) algorithm [30], which is well-known for its good performance with continuous data. We used the implementation provided in the Python library Scikit-learn version 0.24.2.

In order to evaluate the quality of these models, we estimated their LL and BIC scores. As indicated by the Supplementary Table S1, the scores of the single-partition clustering models (LCM, GMM and uk-DB) were similar between them, but far from the majority of the multi-partition clustering models (GEAST and GLSL). MPMM was the exception, returning the worst model. In terms of score, GLSL returned the best model, showing the highest BIC score. Although GEAST obtained a better LL, the BIC difference indicated that GEAST probably suffered from overfitting. This intuition was corroborated by the fact that the GEAST model had 18 partitions and 301 parameters, while the GLSL result had 9 partitions and 131 parameters.

In terms of clustering quality, we observed remarkable differences between single-partition clustering models, and even greater differences between single-partition and multi-partition clustering models. First, we observed that both uk-DB (see Supplementary Figure S4) and GMM (see Supplementary Figure S5) were only able to find two subtypes. These two subtypes could be easily associated with the general severity of all the disease symptoms. One subtype was formed by patients with low (mean of 0.06) symptom severity and the other subtype was formed by patients with a slightly higher (mean of 0.18) symptom severity.

The other single-partition clustering model, the LCM (see Supplementary Figure S6), was able to find five subtypes: (i) patients with almost no severity in both motor and non-motor symptoms (mean of 0.05); (ii) patients with slight severity in both motor and non-motor (mean of 0.11), (iii) patients that also had slight severity in both motor and non-motor (mean of 0.12), but with some differences, such as the presence of sweating and the absence of weight loss problems; (iv) patients with slightly higher symptom severity in both motor and non-motor symptoms (mean of 0.17), especially in mental problems such as depression, apathy and anxiety; (v) patients with mild severity in all symptoms (mean of 0.25).

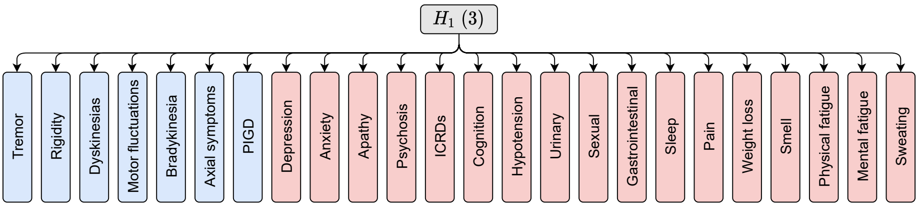

SUPPLEMENTARY FIGURE S4: Bayesian network structure of the single-partition clustering model returned by the uk-DB algorithm. Colors and parentheses have the same meaning as in Figure 1.

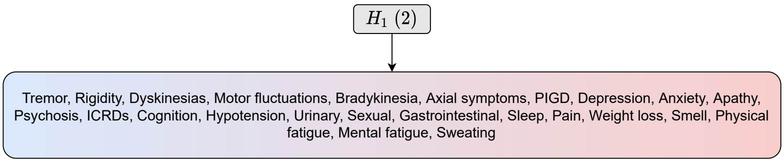

SUPPLEMENTARY FIGURE S5: Bayesian network structure of the single-partition clustering model returned by the GMM algorithm. Colors and parentheses have the same meaning as in Figure 1. We used the style of Poon et al. [1] to represent joint nodes (i.e., nodes with a set of observed variables).

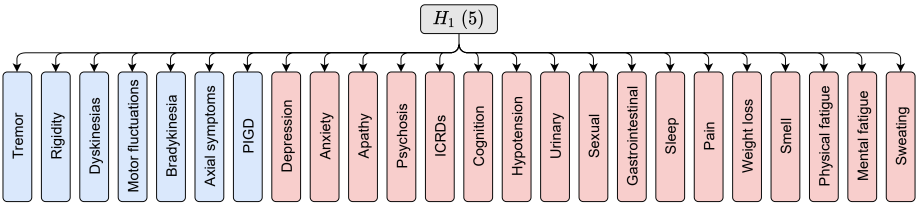

SUPPLEMENTARY FIGURE S6: Bayesian network structure of the single-partition clustering model returned by the LCM algorithm. Colors and parentheses have the same meaning as in Figure 1.

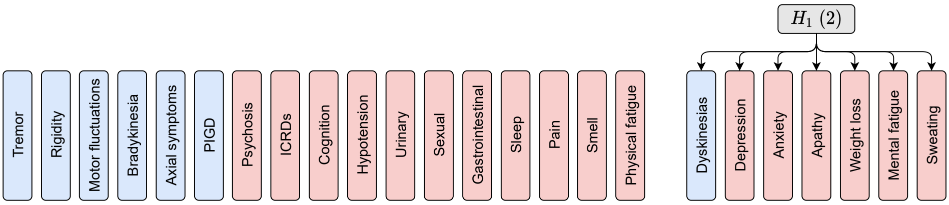

SUPPLEMENTARY FIGURE S7: Bayesian network structure of the multi-partition clustering model returned by the MPMM algorithm. Colors and parentheses have the same meaning as in Figure 1.

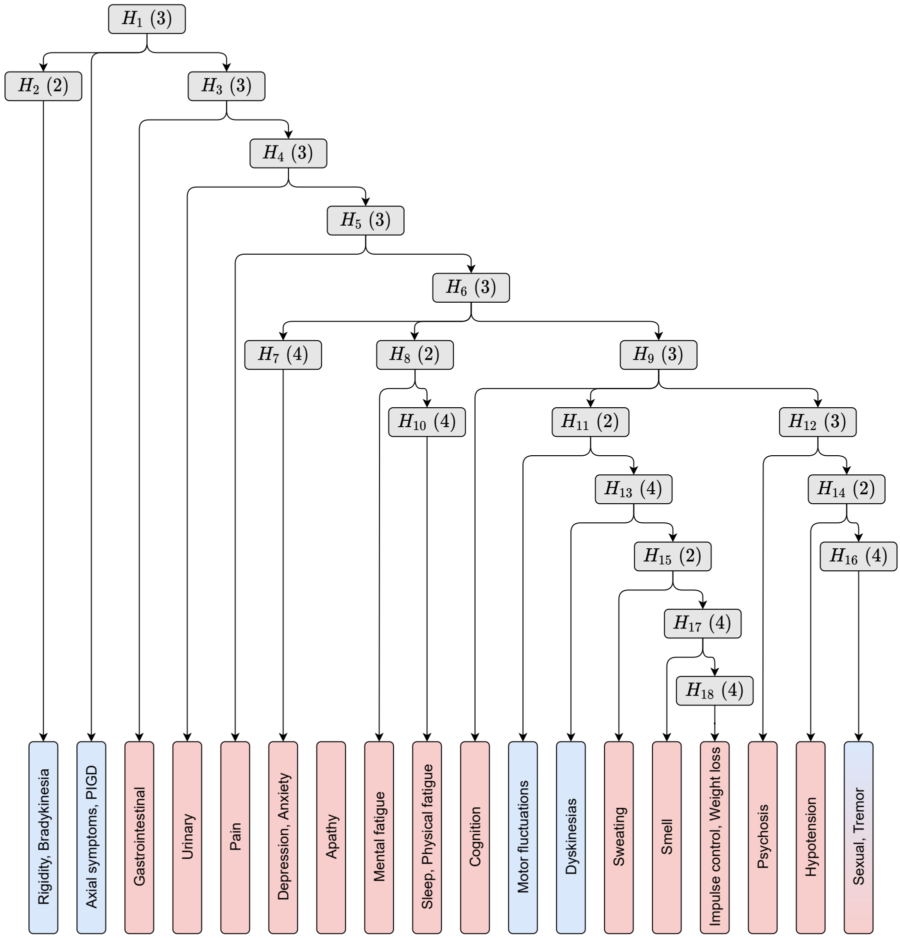

SUPPLEMENTARY FIGURE S8: Bayesian network structure of the multi-partition clustering model returned by the GEAST algorithm. Colors and parentheses have the same meaning as in Figure 1. We used the style of Poon et al. [1] to represent joint nodes (i.e., nodes with a set of observed variables).

While LCM was able to find more subtypes than both GMM and uk-DB, its subtypes also lacked specificity. The absence of feature selection or multiple partitions led to every subtype being dependent on all the motor and non-motor symptoms. This resulted in insubstantial variables (i.e., tremor and rigidity, whose severity did not change much between subtypes) and general patterns (i.e., there were no remarkable differences between subtypes apart from subtypes 1 and 5, which were practically opposite). The main problem of single-partition clustering models lies on the dimensionality of each subtype. Given that each subtype depends on all of the data attributes, increasing the number of subtypes to model a specific pattern results in a large number of unnecessary parameters (overfitting). For this reason, and to avoid overfitting, single-partition clustering models usually end up identifying only a few subtypes.

Multi-partition clustering models returned by GEAST (see Supplementary Figure S8) and GLSL (see Figure 1) were able to find a higher number of subtypes that were only related to certain data attributes. These subtypes were not only far more specific, but they also appeared to be far more faithful to the data (much higher scores). Between the two algorithms, GLSL was able to find the highest scoring model. While GEAST was able to find a good-fitting model, its intrinsic restriction to learning tree structures resulted in a model with 18 partitions (55 subtypes) that was difficult to interpret. Alternatively, the model found by GLSL had 9 partitions, with a total of 19 subtypes. In addition, it was able to exclude the tremor and rigidity symptoms from the clustering, which, as previously commented, appeared to be independent of the rest of symptoms. Compared to the GEAST result, GLSL returned a model much easier to interpret.

4.2 Statistical tests

In this section, we provide the results (i.e., p-values) of the statistical tests that were performed in partition analysis. See Supplementary Tables S2 and S3.

4.3 Probabilistic inference

A total of 29 probabilistic queries were performed to analyze the connections between the 8 partitions. See Supplementary Table S4.

SUPPLEMENTARY TABLE S2

Differences in sex, age, age of onset, disease duration and H&Y stage of subtypes (p-values)

	Subtypes	Sex	Age	Age at onset	Duration	H&Y
Partition A	$A1$ - $A2$	0.96	**0.007**	**< 0.001**	0.06	0.24
Partition B	$B1$ - $B2$	0.64	**0.003**	0.40	**< 0.001**	**< 0.001**
Partition C	$C1$ - $C2$	0.76	0.90	**0.006**	**0.001**	**< 0.001**
	$C1$ - $C3$	0.76	0.90	**0.006**	**0.001**	**0.009**
	$C2$ - $C3$	0.76	0.90	0.90	0.46	0.95
Partition D	$D1$ - $D2$	0.89	0.01	**< 0.001**	**0.007**	0.06
Partition E	$E1$ - $E2$	0.72	**0.006**	**< 0.001**	**< 0.001**	**< 0.001**
Partition F	$F1$ - $F2$	**0.001**	0.06	0.39	**0.003**	**< 0.001**
Partition G	$G1$ - $G2$	0.55	0.43	0.35	0.13	0.27
Partition H	$H1$ - $H2$	0.40	**< 0.001**	**< 0.001**	**0.009**	0.37

Significant differences ($p < 0.01$) are indicated in bold.

Abbreviations: H&Y, Hoehn and Yahr scale.

SUPPLEMENTARY TABLE S3

Differences in Levodopa and DA treatments of subtypes (p-values)

	Subtypes	Levodopa (% medicated)	LDD (mg)	DA (% medicated)	LEDD-DA (mg)
Partition A	$A1$ - $A2$	0.33	**0.002**	0.03	0.36
Partition B	$B1$ - $B2$	**0.004**	**< 0.001**	0.01	0.06
Partition C	$C1$ - $C2$	**< 0.001**	**0.001**	0.84	0.48
	$C1$ - $C3$	0.11	**0.001**	0.84	0.48
	$C2$ - $C3$	0.38	**0.001**	0.84	0.48
Partition D	$D1$ - $D2$	0.01	**< 0.001**	0.59	0.08
Partition E	$E1$ - $E2$	**< 0.001**	**< 0.001**	**0.002**	0.24
Partition F	$F1$ - $F2$	0.09	**0.002**	0.42	0.48
Partition G	$G1$ - $G2$	0.03	**0.008**	0.27	**0.009**
Partition H	$H1$ - $H2$	0.14	**0.001**	0.80	0.01

Significant differences ($p < 0.01$) are indicated in bold.

SUPPLEMENTARY TABLE S4
Probabilistic queries that were performed to analyze the associations between the partitions identified by GLSL.

Probabilistic query	Probability increase	Result
$P(B \mid A = A1)$	$B1$: +0.05	$0.49 \rightarrow 0.54$
$P(B \mid A = A2)$	$B2$: +0.24	$0.51 \rightarrow 0.75$
$P(A \mid B = B1)$	$A1$: +0.08	$0.84 \rightarrow 0.92$
$P(A \mid B = B2)$	$A2$: +0.08	$0.16 \rightarrow 0.24$
$P(C \mid B = B1)$	$C1$: +0.19	$0.59 \rightarrow 0.78$
$P(C \mid B = B2)$	$C2$: +0.10	$0.27 \rightarrow 0.37$
	$C3$: +0.09	$0.14 \rightarrow 0.23$
$P(B \mid C = C1)$	$B1$: +0.16	$0.49 \rightarrow 0.65$
$P(B \mid C = C2)$	$B2$: +0.16	$0.51 \rightarrow 0.67$
$P(B \mid C = C3)$	$B2$: +0.37	$0.51 \rightarrow 0.88$
$P(D \mid B = B1)$	$D1$: +0.20	$0.66 \rightarrow 0.86$
$P(D \mid B = B2)$	$D2$: +0.20	$0.34 \rightarrow 0.53$
$P(B \mid D = D1)$	$B1$: +0.14	$0.49 \rightarrow 0.63$
$P(B \mid D = D2)$	$B2$: +0.28	$0.51 \rightarrow 0.79$
$P(F \mid B = B1)$	$F1$: +0.22	$0.55 \rightarrow 0.77$
$P(F \mid B = B2)$	$F2$: +0.22	$0.45 \rightarrow 0.67$
$P(B \mid F = F1)$	$B1$: +0.19	$0.49 \rightarrow 0.68$
$P(B \mid F = F2)$	$B2$: +0.23	$0.51 \rightarrow 0.74$
$P(G \mid B = B1)$	$G1$: +0.11	$0.80 \rightarrow 0.91$
$P(G \mid B = B2)$	$G2$: +0.11	$0.20 \rightarrow 0.30$
$P(B \mid G = G1)$	$F1$: +0.06	$0.49 \rightarrow 0.55$
$P(B \mid G = G2)$	$F2$: +0.29	$0.51 \rightarrow 0.80$
$P(E \mid D = D1)$	$E1$: +0.10	$0.47 \rightarrow 0.57$
$P(E \mid D = D2)$	$E2$: +0.20	$0.53 \rightarrow 0.73$
$P(D \mid E = E1)$	$D1$: +0.14	$0.66 \rightarrow 0.80$
$P(D \mid E = E2)$	$D2$: +0.13	$0.34 \rightarrow 0.47$
$P(H \mid G = G1)$	$H1$: +0.05	$0.74 \rightarrow 0.79$
$P(H \mid G = G2)$	$H2$: +0.18	$0.26 \rightarrow 0.44$
$P(G \mid H = I1)$	$G1$: +0.05	$0.80 \rightarrow 0.85$
$P(G \mid H = I2)$	$G2$: +0.13	$0.34 \rightarrow 0.47$

References

[1] Poon, L. K., Zhang, N. L., Liu, T. & Liu, A. H. Model-based clustering of high-dimensional data: Variable selection versus facet determination. *Int. J. Approx. Reason.* **54**(1), 196-215 (2013).

[2] Galimberti, G., Manisi, A. & Soffritti, G. Modelling the role of variables in model-based cluster analysis. *Stat. Comput.* **28**(1), 145-169 (2018).

[3] Li, X., Chen, Z., Poon, L. K. & Zhang, N. L. Learning latent superstructures in variational autoencoders for deep multidimensional clustering. In *Proceedings of the 7th International Conference on Learning Representations (ICLR)* 1-15 (2019).

[4] Falck, F. *et al.* Multi-facet clustering variational autoencoders. Preprint at https://arxiv.org/abs/2106.05241 (2021).

[5] McLachlan, G. J., Lee, S. X. & Rathnayake, S. I. Finite mixture models. *Annu. Rev. Stat. Appl.* **6**(1), 355-378 (2019).

[6] Pearl, J. *Probabilistic Reasoning in Intelligent Systems: Networks of Plausible Inference*. (Morgan Kaufmann, 1988).

[7] Koller, D. & Friedman, N. *Probabilistic Graphical Models: Principles and Techniques*. (The MIT Press, 2009).

[8] Lauritzen, S. L. & Wermuth, N. Graphical models for associations between variables, some of which are qualitative and some quantitative. *Ann. Stat.* **17**, 31–57 (1989).

[9] Scanagatta, M., Salmerón, A. & Stella, F. A survey on Bayesian network structure learning from data. *Prog. Artif. Intell.* **8**, 425-439 (2019).

[10] Akaike, H. A new look at the statistical model identification. *IEEE Trans. Automat. Contr.* **19**(6), 716-723 (1974).

[11] Schwarz, G. Estimating the dimension of a model. *Ann. Stat.* **6**(2), 461-464 (1978).

[12] Friedman, N. Learning belief networks in the presence of missing values and hidden variables. In *Proceedings of the 14th International Conference on Machine Learning (ICML)* 125-133 (1997).

[13] Dempster, A. P., Laird N. M. & Rubin, D. B. Maximum likelihood from incomplete data via the EM algorithm. *J. R. Stat. Soc. (Series B Stat. Methodol.)* **39**(1), 1-38 (1977).

[14] Kim, J. & Pearl, J. A computational model for causal and diagnostic reasoning in inference systems. In *Proceedings of the 7th International Joint Conference on Artificial Intelligence (JCAI)* 190-193 (1983).

[15] Lerner, U. & Parr, R. Inference in hybrid networks: Theoretical limits and practical algorithms. In *Proceedings of the 17th Conference on Uncertainty in Artificial Intelligence (UAI)* 310-318 (2001).

[16] Salmeron, A., Rumi, R., Lanseth, H., Nielsen, D. & Madsen, A. L. A review of inference algorithms for hybrid Bayesian networks. *J. Artif. Intell. Res.* **62**, 799-828 (2018).

[17] Attias, H. A variational Bayesian framework for graphical models. In *Proceedings of the 14th Conference on Neural Information Processing Systems (NIPS)* 209-215 (2000).

[18] Hastings, W. K. Monte Carlo sampling methods using Markov chains and their applications. *Biometrika* **57**(1), 97-109 (1970).

[19] Blei, D. M., Kucukelbir, A. & McAuliffe, J. D. Variational inference: A review for statisticians. *J. Am. Stat. Assoc.* **112**(518), 859-877 (2016).

[20] Winn, J. & Bishop, C. M. Variational message passing. *J. Mach. Learn. Res.* **6**, 661-694 (2005).

[21] Chickering, D., Geiger, D. & Heckerman, D. Learning Bayesian networks: Search methods and experimental results. In *Proceedings of the 5th International Workshop on Artificial Intelligence and Statistics (AISTATS)* 112-128 (1995).

[22] Beal, M. J. & Ghahramani, Z. The variational Bayesian EM algorithm for incomplete data: With application to scoring graphical model structures. *Bayesian Stat.* **7**(210), 453-464 (2003).

[23] Benjumeda, M., Luego-Sanchez, S., Larrañaga, P. & Bielza, C. Tractable learning of Bayesian networks from partially observed data. *Pattern Recognit.* **91**, 190-199 (2019).

[24] Kwisthout, J., Boadlaender, H. L. & van der Gaag L. C. The necessity of bounded treewidth for efficient inference in Bayesian networks. In *Proceedings of the 19th European Conference on Artificial Intelligence (ECAI)* 237-242 (2010).

[25] Bishop, C. M. *Pattern Recognition and Machine Learning*. (Springer, 2016).

[26] Casella, G. An introduction to empirical Bayes data analysis. *Am. Stat.* **39**(2), 83-87 (1985).

[27] Rodriguez-Sanchez, F., Larrañaga, P. & Bielza, C. Incremental learning of latent forests. *IEEE Access* **8**, 224420-224432 (2020).

[28] Lazarsfeld, P. F. & Henry, N. W. *Latent Structure Analysis*. (Houghton & Mifflin, 1968).

[29] Pham, D. T. & Ruz, G. A. Unsupervised training of Bayesian networks for data clustering. *Proc. R. So.c Lond. A Math. Phys. Sci.* **465**(2109), 2927-2948 (2009).

[30] Azur, M. J., Stuart, E. A., Frangakis, C. & Leaf, P. J. Multiple imputation by chained equations: What is it and how does it work?. *Int. J. Methods Psychiatr. Res.* **20**(1), 40-49 (2011).
